# Selective C-Arylation of 2,5-Dibromo-3-hexylthiophene via Suzuki Cross Coupling Reaction and Their Pharmacological Aspects

**DOI:** 10.3390/molecules20035202

**Published:** 2015-03-23

**Authors:** Hafiz Mansoor Ikram, Nasir Rasool, Gulraiz Ahmad, Ghayoor Abbas Chotana, Syed Ghulam Musharraf, Muhammad Zubair, Usman Ali Rana, Muhammad Zia-Ul-Haq, Hawa Ze Jaafar

**Affiliations:** 1 Department of Chemistry, Government College University Faisalabad, Faisalabad 38000, Pakistan; E-Mails: chemistue@gmail.com (H.M.I.); gulchemist35@gmail.com (G.A.); nr_308@hotmail.com (M.Z.); 2 Department of Chemistry, SBA School of Science & Engineering, Lahore University of Management Sciences, Sector U, DHA, Lahore Cantt. 54792, Pakistan; E-Mail: ghayoor.abbas@lums.edu.pk; 3 International Center for Chemical and Biological Sciences, HEJ Research Institute of Chemistry, University of Karachi, Karachi 75270, Pakistan; E-Mail: musharraf1977@yahoo.com; 4 Sustainable Energy Technologies (SET) center, College of Engineering, PO-Box 800, King Saud University, Riyadh 11421, Saudi Arabia; E-Mail: urana@ksu.edu.sa; 5 The Patent Office, Karachi 74200, Pakistan; E-Mail: ahirzia@gmail.com; 6 Department of Crop Science, Faculty of Agriculture, University Putra Malaysia, Serdang 43400, Selangor, Malaysia

**Keywords:** Palladium(0), 2,5-dibromo-3-hexylthiophene, biofilm inhibition, hemolysis assay, anti-thrombolytic assay

## Abstract

The present study reports the synthesis of various new derivatives based on 5-aryl-2-bromo-3-hexylthiophene with moderate-to-good yields via a palladium-catalyzed Suzuki cross-coupling reaction. This coupling method involved the reaction of 2,5-dibromo-3-hexylthiophene with several arylboronic acids in order to synthesize corresponding thiophene derivatives under controlled and optimal reaction conditions. The different substituents (CH_3_, OCH_3_, Cl, F *etc.*) present on arylboronic acids are found to have significant electronic effects on the overall properties of new products. The synthesized thiophene molecules were studied for their haemolytic, biofilm inhibition and anti-thrombolytic activities, and almost all products showed potentially good properties. The compound 2-bromo-5-(3-chloro-4-fluorophenyl)-3-hexylthiophenein particular exhibited the highest values for haemolytic and bio-film inhibition activities among all newly synthesized derivatives. In addition, the compound 2-bromo-3-hexyl-5-(4-iodophenyl)thiophene also showed high anti-thrombolytic activity, suggesting the potential medicinal applications of these newly synthesized compounds.

## 1. Introduction

In the area of synthetic organic chemistry, Suzuki cross-coupling reactions have emerged as an efficient and remarkably effective method for carbon-carbon bond formation [1]. Hence, the coupling reactions of weakly basic heterocyclic compounds with organoboron compounds *i.e.*, arylboronic acids and esters, catalyzed by palladium have been found to be very useful for the synthesis of advanced materials, agrochemicals and pharmaceutical compounds at both the laboratory and industrial levels [2]. The Suzuki cross-coupling reaction is largely preferred over other types of coupling reactions because it can be carried out under normal reaction conditions with a wide range of functional group tolerance, it is unaffected by aqueous solvent, and it generates non-toxic by-products which can be separated easily [3]. Substituted thiophene compounds are present in various natural and biologically active compounds [4]. Thiophene based molecules have a great deal of importance and exhibit medicinal activities such as anti-fungal, anti-inflammatory, anti-microbial, analgesic, anti-urease, anti-tubercular, anti-depressant, BASE1 inhibitors, anti-HIV PR inhibitors and anti-breast cancer activities [4,5]. In addition, thiophene compounds have great applications in electrochromic devices [6], non-linear optics [7], azo dyes [4], energy storage devices [8], conductivity-based sensors, biodiagnostics, optoelectronics systems and superconductors [4]. In order to investigate an easy and facile scheme to make thiophene-based derivates, the aim of the present study was to synthesize different derivatives of 2,5-dibromo-3-hexylthiophene using different arylboronic acids via palladium-catalyzed Suzuki cross-coupling reactions. In addition to the synthesis, we also investigated the biofilm inhibition, hemolytic and anti-thrombolytic activities of these thiophene derivatives, and the results revealed potentially high activity from these newly synthesized thiophene derivatives against several diseases.

## 2. Results and Discussion

### 2.1. Chemistry

**Scheme 1:**
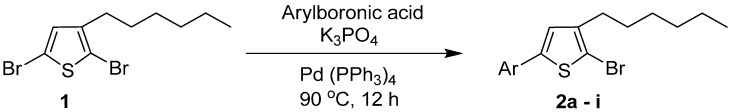
Synthesis of 5-aryl-2-bromo-3-hexylthiophene (**2a**–**i**). *Reagents and conditions*: 1 (1 mmol), Arylboronic acids (1 mmol), K_3_PO_4_ (1.75 mmol), Pd(PPh_3_)_4_ (4 mol %), solvent/H_2_O (4:1), (see Table 1), 90 °C, 12 h.

The term “regioselectivity” refers to the greater reactivity of a carbon moiety (due to being more electron-deficient) towards nucleophiles, whereas other carbons being reactive do not display any response to the attacking nucleophile. This term can be applied to palladium-cataylzed cross-coupling reactions of different substrates [9]. Furan, thiophene and pyrrole undergo electrophilic substitution reactions such as halogenation reactions. In these substitution reactions, the lone pair of electrons belonging to O, N and S is donated to the ring. However, when these halogenated thiophenes undergo palladium-catalyzed cross-coupling reactions with high oxidative addition, the arylboronate ion preferably attacks the carbon, which is deficient in electrons due to being bonded with the halogen. It was also noted that the transmetallation process is faster due to negatively charged arylboronate ion than the neutral boronic acid [10]. Dang *et al.* [11] reported the Suzuki cross-coupling reaction involving tetrabromothiophene and arylboronic acids to synthesize tetraarylthiophenes. Herein, we have explored the application of Suzuki cross-coupling reactions [12] to synthesize new thiophene derivatives. To the best of our knowledge, Suzuki cross-coupling reactions of 2,5-dibromo-3-hexylthiophene derivatives have not been investigated so far. The reactions were carried out at 90 °C temperature to get moderate-to-good yields of the desired products. In the current research work, Pd(PPh_3_)_4_was used as catalyst and K_3_PO_4_ was used as a base. The Suzuki reaction of 1(1 mmol) with different arylboronic acids (1 mmol) resulted in5-aryl-2-bromo-3-hexylthiophenes (**2a**–**i**) in moderate to good yields (Scheme 1, Table 1). The results from the present study revealed that greater yields of products were obtained upon using 1,4-dioxane as solvent as compared to toluene, probably due to relatively higher solubility of arylboronic acids in 1,4-dioxane. The high boiling point of toluene makes it beneficial for reactions carried out at high temperature, but its negative impact is poor yields of products. We found that the yield of the final product was significantly affected by the solvent [13]. A number of different 5-aryl-2-bromo-3-hexylthiophene derivatives (**2a**–**i**) (Table 1) were synthesized by using a wide range of arylboronic acids. Different derivatives of 2,5-dibromo-3-hexylthiophene were synthesized (Scheme 1) and the desired products (**2a**–**i**) were attained in moderate–to-good yields when 1,4-dioxane was used as a solvent (entries 1–9). Only moderate yields were obtained when toluene was used as solvent. The final yields of the products were greatly influenced by the conditions such as temperature, nature of solvent and the water content. The best solvent/water ratio was found to be 4:1 (solvent/water), as reported by [14].

**Table 1:** Synthesis of 5-aryl-2-bromo-3-hexylthiophene (**2a**–**i**).

Entry	Aryl Boronic Acids	Product	Solvent/H_2_O (4:1)	Yield%
1	4-MeC_6_H_4_B(OH)_2_	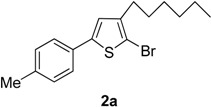	Dioxane	76
			Toluene	57
2	3,5-Me_2_C_6_H_3_B(OH)_2_	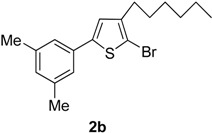	Dioxane	70
3	4-MeOC_6_H_4_B(OH)_2_	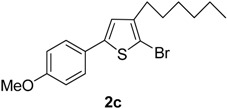	Dioxane	68
4	4-ClC_6_H_4_B(OH)_2_	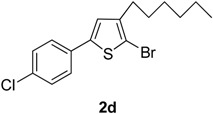	Dioxane	77
5	4-IC_6_H_4_B(OH)_2_	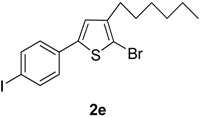	Dioxane	69
6	3,5-F_2_C_6_H_3_B(OH)_2_	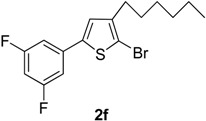	Dioxane	65
7	3-Cl,4-FC_6_H_3_B(OH)_2_	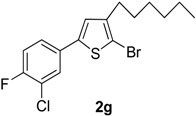	Dioxane	68
8	4-MeSC_6_H_4_B(OH)_2_	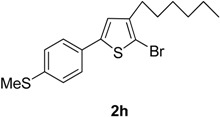	Dioxane	62
9	3-AcC_6_H_4_B(OH)_2_	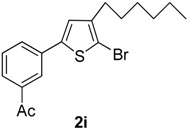	Dioxane	72

### 2.2. Pharmacological Aspects

#### 2.2.1. Haemolytic Activity

**Figure 1:**
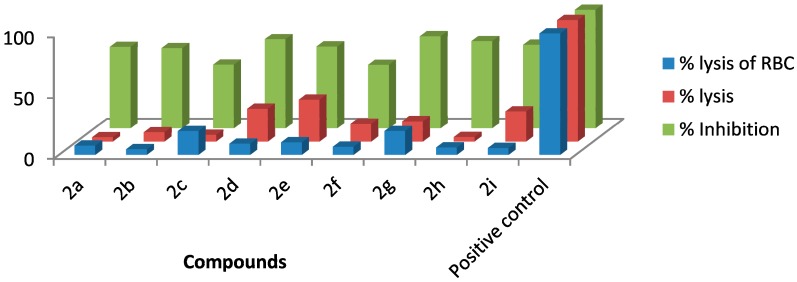
Values of haemolytic, anti-thrombolytic and biofilm inhibition activities.

Compared with the positive control triton X-100 standard, compounds **2a**–**b**, **2f** and **2h**–**i** showed moderate haemolytic activity, whereas compounds **2c** and **2g** exhibited the highest toxicity effects. It was surprisingly noted that compound **2g** exhibited the highest % lysis of RBCs (19.54%) against triton X-100, which might be due to the presence of electron-withdrawing F and Cl moieties present on the phenyl ring. Table 2 shows that compound **2e** showed moderate haemolytic activity (10.22%), probably due to the presence of a bulky iodine group at para position in the aromatic phenyl ring. The data from Table 2 also shows that compounds **2b** and **2i** exhibited low toxicity effect with % lysis values of RBC (4.54%) and (5.51%), respectively. In contrast, compound **2c** showed a higher toxicity value (19.50%). This might be due to the presence of one -OCH_3_ group (negative inductive effect and positive resonance effect). Mologni *et al.* [15] reported that anticancer activity is usually enhanced by the presence of electron-releasing groups (positive resonance effect) [15]. Therefore, the presence of a -OCH_3_ group in compound **2c** could be the possible cause of the higher % lysis of RBCs. In view of the observed differences in the % lysis values of RBC, it is suggested that the electron withdrawing and electron donating functional groups have an influence on the haemolytic activity of the compounds. Ding *et al.* [16] reported that the compounds with the Cl functional group at the benzene ring exhibited better and higher haemolytic activities than the compounds with -CH_3_ substitution at the same position. For this reason, compound **2d** (9.13%) showed better haemolytic activity value than compound **2a** (7.52%). These compounds (**2a**–**i**) showed moderate to high % lysis of RBC and can be used as potential anticancer agents (Figure 1).

**Table 2:** Haemolytic activity data of 5-aryl-2-bromo-3-hexylthiophene based compounds (**2a**–**i**).

Entry	% Lysis of RBC ± SD
**2a**	7.52 ± 0.042
**2b**	4.54 ± 0.054
**2c**	19.50 ± 0.079
**2d**	9.13 ± 0.113
**2e**	10.22 ± 0.084
**2f**	6.55 ± 0.071
**2g**	19.54 ± 0.095
**2h**	5.97 ± 0.102
**2i**	5.51 ± 0.089
Positive Control	100%

#### 2.2.2. Anti-Thrombolytic Activity

Thrombophilia and nephritic syndrome causes a common central nervous disease cerebral venous sinus thrombosis (CVST) [17]. For the treatment of CVST, intravenous heparin and warfarin have been explored as anti-coagulant agent, primarily due to their high efficiency [18]. At present, thrombolytic drugs have been used as an alternative cure for this fatal disease [19]. Several thrombolytic agents like streptokinase and urokinase are playing a vital role in the treatment of cerebral venous sinus thrombosis [20]. The compounds **2e**, **2d** and **2i** exhibited better thrombolytic effects, while compounds **2a**–**c** and **2h** showed mild to moderate action when compared with the positive control streptokinase standard. Compound **2e** displayed the highest % lysis (34.27) against streptokinase standard, which might be due to the presence of a bulky iodine group at the aryl ring. On the other hand, the compounds **2d** and **2i** (Table 3) showed moderate activity values of 26.75% and 24.59%, respectively, probably due to the presence of Cl and acetyl groups at the para position of the phenyl ring. The data from Table 3 also shows that the compounds **2a** and **2h** exhibited low effect with % lysis values of 3.43% and 3.60%, respectively. Moreover, it is suggested that the presence of electron-withdrawing groups (negative inductive effect) promote thrombolytic activity, while the position of functional groups is also found to affects the thrombolytic activity of all newly synthesized compounds. These compounds (**2a**–**i**) showed moderate to high % lysis and can be used as potential thrombolytic agents (Figure 1).

**Table 3:** Anti-thrombolytic activity data of 5-aryl-2-bromo-3-hexylthiophene based compounds (**2a**–**i**).

Entry	% Lysis ± SD
**2a**	3.43 ± 0.022
**2b**	7.58 ± 0.031
**2c**	5.43 ± 0.015
**2d**	26.75 ± 0.071
**2e**	34.27 ± 0.019
**2f**	14.54 ± 0.053
**2g**	16.52 ± 0.035
**2h**	3.6 ± 0.027
**2i**	24.59 ± 0.046
Positive control	100

#### 2.2.3. Biofilm Inhibition Assay

Biofilm formation by pathogenic bacteria is an increasing cause of morbidity and mortality associated with several fatal infections. Biofilm is conglomerations of bacterial cells, which is protected by a self-created extra polymeric substance. Biofilms are very difficult to eliminate and cause obstinate infections [21]. The anti-biofilm activity of synthesized thiophene derivatives (**2a**–**i**) was studied against strains of *Escherichia coli*. Table 4 shows the results of biofilm inhibition for all synthesized compounds. It was observed that a higher positive charge density leads to strong electrostatic interaction [22]. Based on the results of antimicrobial activity of the newly synthesized compounds **2g** and **2d** were considerably affected by electron-withdrawing substituents and the incorporation of electron-withdrawing groups (negative inductive effect) responsible for enhancing activity against the test microorganism when compared with standard Rifampicin. Compounds **2d**, **2e** and **2g**, which contain electron-withdrawing functional groups, showed good inhibition values (73.13%, 67.02%, and 75.66%, respectively) against *E. coli*. Compound **2c**, which contains electron-donating methyl groups, showed low biofilm inhibitory activity (52.12%) against *E. coli*. Surprisingly, it was observed that compound **2h**, which has a negative inductive and positive resonance effect, exhibited good biofilm inhibition (71.52%) against *E. coli*.

**Table 4:** Biofilm inhibition activity data of 5-aryl-2-bromo-3-hexylthiophene based compounds (**2a**–**i**).

Entry	% Inhibition ± SD
**2a**	66.75± 0.154
**2b**	65.69± 0.134
**2c**	52.12± 0.165
**2d**	73.13± 0.154
**2e**	67.02± 0.167
**2f**	51.99± 0.188
**2g**	75.66± 0.176
**2h**	71.52± 0.163
**2i**	68.48± 0.139
Positive control	97.43

## 3. Experimental

### 3.1. General

A Bruker Aspect AM-400 NMR instrument was used to study ^1^H-NMR and ^13^C-NMR spectroscopic values in CDCl_3_ and CD_3_OD at 400/100 MHz. The chemical shift values are reported in δ ppm and the coupling constant values in Hertz (Hz). The melting points of newly synthesized products were obtained from a Buchi B-540 melting point apparatus. For synthesis, all analytical chemicals were obtained from Alfa Aesar and Sigma Aldrich. EI-MS values of the products were obtained from data system containing JMS-HX-110 spectrometer. To assist column chromatography, both types of silica gels 70–230 mesh and 230–400 mesh were used. For proper monitoring of the reaction, the Merck silica gel 60 PF_254_ TLC cards were used. The new compounds were investigated by UV lamp of wavelength 254 to 365 nm.

### 3.2. General Procedure for the Synthesis of 5-Aryl-2-Bromo-3-Hexylthiophene (**2a**–**i**)

The 2,5-dibromo-3-hexylthiophene (1 mmol) and 4 mol % Pd(PPh_3_)_4_ were added in an organic solvent 1,4-dioxane (2 mmol) under inert (argon) atmosphere. The mixture was stirred in a Schlenk flask at 25 °C for a duration of 30 min. After stirring, the arylboronic acids (1 mmol) and K_3_PO_4_ (1.75 mmol) were added along with water (0.5 mL) under the same atmosphere. The solution was stirred again at 90 °C for a duration of 12 h. After stirring for 12 h, the reaction mixture was cooled to room temperature. The organic layer in the reaction mixture was separated by using CH_3_COOC_2_H_5_ and desiccated above MgSO_4_. After that, the excess solvent was evaporated by applying reduced pressure on a rotary evaporator. The crude product thus obtained, and was purified using column chromatography. In chromatography, ethyl acetate and *n*-hexane were used in equal ratios to acquire the final compounds. The end products were characterized by different spectroscopic techniques [23].

### 3.3. Characterization Data

*2-Bromo-3-hexyl-5-(4-methylphenyl)thiophene* (**2a**). Mp: 170–173 °C; ^1^H-NMR (CDCl_3_ + CD_3_OD): δ = 7.70 (d, *J* = 8.05 Hz, 2H-aryl), 7.30 (d, *J* = 8.05 Hz, 2H-aryl), 2.35 (s, 3H-CH_3_), 6.80 (s, 1H-Thiophene), 2.65 (t, *J* = 7.83 Hz, 2H-Methylene), 1.35 (m, 6H-Methylene), 1.30 (m, 2H-Methylene), 0.85 (t, *J* = 6.08 Hz, 3H-CH_3_). ^13^C-NMR (CDCl_3_ + CD_3_OD): δ = 14.23, 21.11, 22.94, 27.50, 29.01, 31.11, 31.98, 110.10, 125.72, 126.80, 129.70, 130.87, 131.90, 141.43, 142.10. EIMS (*m/z* + ion mode): 337.32: [M-Br]^+^ = 257.42: [M-C_6_H_13_]^+^ = 172.42: [M-Thiophene]^+^ = 91.42: [M-CH_3_]^+^ = 76.15. Anal. Calcd. for C_17_H_21_BrS (337.32): C, 60.53; H, 6.28. Found: C, 60.41; H, 6.17%.

*2-Bromo-3-hexyl-5-(3,5-dimethylphenyl)thiophene* (**2b**). Mp: 175–177 °C; ^1^H-NMR (CDCl_3_ + CD_3_OD): δ = 7.60 (s, 2H-aryl), 7.35 (s, 1H-aryl), 2.35 (s, 6H-2CH_3_), 6.90 (s, 1H-Thiophene), 2.62 (t, *J* = 7.61 Hz, 2H-Methylene), 1.32 (m, 6H-Methylene), 1.28 (m, 2H-Methylene), 0.88 (t, *J* = 6.20 Hz, 3H-CH_3_). ^13^C-NMR (CDCl_3_ + CD_3_OD): δ = 14.21, 21.98, 22.89, 27.38, 28.99, 31.19, 32.01, 110.15, 127.13, 127.51, 130.99, 133.59, 138.90, 141.40, 142.05. EIMS (*m/z*, + ion mode): 351.34: [M-Br]^+^ = 271.44: [M-C_6_H_13_]^+^ = 186.44: [M-Thiophene]^+^ = 105.44: [M-2CH_3_]^+^ = 75.40. Anal. Calcd. for C_18_H_23_BrS (351.34): C, 61.53; H, 6.60. Found: C, 61.43; H, 6.51%.

*2-Bromo-3-hexyl-5-(4-methoxyphenyl)thiophene* (**2c**). Mp: 174 °C; ^1^H-NMR (CDCl_3_ + CD_3_OD): δ = 7.50 (d, *J* = 8.28 Hz, 2H-aryl), 7.05 (d, *J* = 6.18 Hz, 2H-aryl), 6.85 (s, 1H-Thiophene), 3.83 (s, 3H-OCH_3_), 2.60 (t, *J* = 7.71 Hz, 2H-Methylene), 1.33 (m, 6H-Methylene), 1.28 (m, 2H-Methylene), 0.90 (t, *J* = 6.23 Hz, 3H-CH_3_). ^13^C-NMR (CDCl_3_ + CD_3_OD): δ = 14.15, 22.84, 27.49, 29.07, 31.15, 31.85, 55.89, 110.13, 114.71, 126.08, 127.11, 128.61, 141.28, 142.06. 160.67. EIMS (*m/z*, + ion mode): 353.32: [M-Br]^+^ = 273.42: [M-C_6_H_13_]^+^ = 188.42: [M-Thiophene]^+^ = 107.42: [M-OCH_3_]^+^ = 76.20. Anal. Calcd. for C_17_H_21_BrOS (353.32): C, 57.79; H, 5.99. Found: C, 57.68; H, 5.91%.

*2-Bromo-3-hexyl-5-(4-chlorophenyl)thiophene* (**2d**). Mp: 175 °C; ^1^H-NMR (CDCl_3_ + CD_3_OD): δ = 7.60 (d, *J* = 7.91 Hz, 2H-aryl), 7.75 (d, *J* = 6.01 Hz, 2H-aryl), 6.88 (s, 1H-Thiophene), 2.63 (t, *J* = 7.69 Hz, 2H-Methylene), 1.31 (m, 6H-Methylene), 1.27 (m, 2H-Methylene), 0.86 (t, *J* = 6.16 Hz, 3H-CH_3_). ^13^C-NMR (CDCl_3_ + CD_3_OD): δ = 14.12, 22.91, 27.47, 28.93, 31.24, 31.94, 110.03, 126.93, 128.88, 129.41, 131.78, 134.43, 141.33, 142.17. EIMS (*m/z*, + ion mode): 357.74: [M-Br]^+^ = 277.84: [M-C_6_H_13_]^+^ = 192.80: [M-Thiophene]^+^ = 111.80: [M-Cl]^+^ = 76.30. Anal. Calcd. for C_16_H_18_BrClS (357.74): C, 53.72; H, 5.07. Found: C, 53.67; H, 5.01%.

*2-Bromo-3-hexyl-5-(4-iodophenyl)thiophene* (**2e**). Mp: 179 °C; ^1^H-NMR (CDCl_3_ + CD_3_OD): δ = 7.62 (d, *J* = 8.18 Hz, 2H-aryl), 8.05 (d, *J* = 6.25 Hz, 2H-aryl), 6.82 (s, 1H-Thiophene), 2.65 (t, *J* = 7.58 Hz, 2H-Methylene), 1.35 (m, 6H-Methylene), 1.29 (m, 2H-Methylene), 0.89 (t, *J* = 6.17 Hz, 3H-CH_3_). ^13^C-NMR (CDCl_3_ + CD_3_OD): δ = 14.08, 22.73, 27.49, 28.81, 31.17, 32.01, 94.37, 110.07, 127.12, 129.14, 132.67, 138.19, 141.31, 142.01. EIMS (*m/z*, + ion mode): 449.19: [M-Br]^+^ = 369.29: [M-C_6_H_13_]^+^ = 284.20: [M-Thiophene]^+^ = 203.20: [M-I]^+^ = 76.32. Anal. Calcd. for C_16_H_18_BrIS (449.19): C, 42.78; H, 4.04. Found: C, 42.69; H, 3.99%.

*2-Bromo-5-(3,5-difluorophenyl)-3-hexylthiophene* (**2f**). Mp: 171–173 °C; ^1^H-NMR (CDCl_3_ + CD_3_OD): δ = 7.35 (s, 2H-aryl), 6.85 (s, 1H-aryl), 6.75 (s, 1H-Thiophene), 2.60 (t, *J* = 7.74 Hz, 2H-Methylene), 1.33 (m, 6H-Methylene), 1.27 (m, 2H-Methylene), 0.90 (t, *J* = 6.19 Hz, 3H-CH_3_). ^13^C-NMR (CDCl_3_ + CD_3_OD): δ = 14.19, 22.79, 27.57, 28.92, 31.18, 31.95, 103.76, 109.16, 111.78, 126.90, 136.73, 140.87, 143.01, 167.02. EIMS (*m/z*, + ion mode): 359.27: [M-Br]^+^ = 279.37: [M-C_6_H_13_]^+^ = 194.34: [M-Thiophene]^+^ = 113.34: [M-2F]^+^ = 75.35. Anal. Calcd. for C_16_H_17_BrF_2_S (359.27): C, 53.49; H, 4.77. Found: C, 53.37; H, 4.68%.

*2-Bromo-5-(3-chloro-4-fluorophenyl)-3-hexylthiophene* (**2g**). Mp: 174 °C; ^1^H-NMR (CDCl_3_ + CD_3_OD): δ = 8.05 (s, 1H-aryl), 7.70 (d, *J* = 7.95 Hz, 1H-aryl), 7.25 (d, *J* = 6.37 Hz, 1H-aryl), 6.85 (s, 1H-Thiophene), 2.65 (t, *J* = 7.68 Hz, 2H-Methylene), 1.37 (m, 6H-Methylene), 1.30 (m, 2H-Methylene), 0.85 (t, *J* = 6.01 Hz, 3H-CH_3_). ^13^C-NMR (CDCl_3_ + CD_3_OD): δ = 14.13, 21.99, 27.12, 28.10, 31.01, 31.56, 110.47, 117.67, 121.52, 127.10, 127.98, 129.87, 130.97, 141.56, 142.77, 158.98. EIMS (*m/z*, +ion mode): 375.73: [M-Br]^+^= 295.83: [M-C_6_H_13_]^+^ = 210.83: [M-Thiophene]^+^ = 129.80: [M-F]^+^ = 110.83: [M-Cl]^+^ = 75.20. Anal. Calcd. for C_16_H_17_BrClFS (375.53): C, 51.15; H, 4.56. Found: C, 51.09; H, 4.53%.

*2-Bromo-3-hexyl-5-(4-(methylthio)phenyl)thiophene* (**2h**). Mp: 175 °C; ^1^H-NMR (CDCl_3_ + CD_3_OD): δ = 7.75 (d, *J* = 8.15 Hz, 2H-aryl), 7.50 (d, *J* = 6.18 Hz, 2H-aryl), 6.88 (s, 1H-Thiophene) 2.55 (s, 3H-SCH_3_), 2.63 (t, *J* = 7.56 Hz, 2H-Methylene), 1.32 (m, 6H-Methylene), 1.28 (m, 2H-Methylene), 0.89 (t, *J* = 6.16 Hz, 3H-CH_3_). ^13^C-NMR (CDCl_3_ + CD_3_OD): δ = 14.04, 14.97, 23.03, 27.23, 28.58, 31.43, 32.17, 109.98, 126.88, 127.05, 128.12, 130.49, 139.65, 141.70, 142.35. EIMS (*m/z*, +ion mode): 369.38: [M-Br]^+^ = 289.40: [M-C_6_H_13_]^+^ = 204.40: [M-Thiophene]^+^ = 123.41: [M-SCH_3_]^+^ = 76.35. Anal. Calcd. for C_17_H_21_BrS_2_ (369.38):C, 55.28; H, 5.73. Found: C, 55.15; H, 5.64%.

*2-Bromo-3-hexyl-5-(3-acetylphenyl)thiophene* (**2i**). Mp: 172 °C; ^1^H-NMR (CDCl_3_ + CD_3_OD): δ = 8.85 (s, 1H-aryl), 8.05 (d, *J* = 7.65 Hz, 1H-aryl), 7.65 (t, *J* = 7.90 Hz, 1H-aryl), 7.95 (d, *J* = 7.43 Hz, 1H-aryl), 6.83 (s, 1H-Thiophene), 2.52 (s, 3H-CH_3_), 2.67 (t, *J* = 7.59 Hz, 2H-Methylene), 1.35 (m, 6H-Methylene), 1.30 (m, 2H-Methylene), 0.92 (t, *J* = 6.08 Hz, 3H-CH_3_). ^13^C-NMR (CDCl_3_ + CD_3_OD): δ = 14.08, 22.81, 26.78, 27.31, 28.59, 31.28, 32.12, 110.17, 126.42, 127.24, 129.04, 129.47, 131.07, 133.73, 137.63, 141.13, 142.30, 198.01. EIMS (*m/z*, +ion mode): 365.33: [M-Br]^+^ = 285.43: [M-C_6_H_13_]^+^ = 200.41: [M-Thiophene]^+^ = 119.41: [M-Ac]^+^ = 76.30. Anal. Calcd. for C_18_H_21_BrOS (365.33): C, 59.18; H, 5.79. Found: C, 59.07; H, 5.73%.

### 3.4. Haemolytic Activity

The haemolytic activity of newly synthesized compounds was studied using the previously reported method [24]. Ethyl acetate was used to make the solution of newly synthesized compounds under study. Approximately 3 mL of newly attained heparinized human blood was gently mixed, poured into a 15 mL sterile Falcon tube and centrifuged at 850 rpm for the period of five minutes. The supernatant was decanted off and the red blood cells were washed three times with ice-cold (4 °C) disinfected isotonic phosphate buffered saline (PBS) pH 7.4 (5 mL) and kept for half an hour at room temperature. The cells were kept in 20 mL ice-cold PBS buffer. Erythrocytes were calculated by heamacytometer, maintained 7.068 × 10^8^ cell per mL and diluted with phosphate buffer saline. About 180 µL suspension of red blood cells was added in the eppendorfs alongwith 20 µL of sample solutions. The tubes were incubated at 37 °C for aperiod of 35 min. After incubation, the tubes were agitated again for 5 min. at 1310 rpm after placing the tubes on ice for the same time. After centrifugation was completed, 100 µL supernatant was diluted with 900 µL ice-cold phosphate buffer saline and samples were placed on ice. About 200 µL solution of each sample was added into 96-well plastic tissue culture plates. 0.1% triton X-100 was used as a positive control for every evaluation, and as a negative control, phosphate buffer saline was used. The absorbance of each sample was observed at 576 nm by Quant instrument.

### 3.5. Biofilm Inhibition Assay

The biofilm inhibition activity of newly synthesized compounds was studied using the method reported in [25]. One hundred micro liters of nutrient broth (Oxoid, UK) alongwith 100 μL sample solutions (**2a**–**i**) and 20 μL of bacterial suspension were kept on the wells of sterile 96-well plastic tissue culture plates. Negative control wells involved only nutrient broth. For 24 h, the plates were incubated at 37 °C after covering. The sterile phosphate buffer (220 μL) was used to wash the content of each well thrice. The non-adherent bacteria were eliminated by shaking the plates vigorously. 220 μL methanol solution with the concentration of 99% was used to fix the remaining bacteria, and after 15 min the empty plates were left to dry. The dried plates were stained with 50% crystal violet (220 mL) for aperiod of 5 min. The plates were washed with tap water for the purpose of removing excess stain. After drying the plates, the dye bound to the adherent cells was resolublized with 220 µL glacial acetic acid 33% per well. The OD of each well was determined at 630 nm by using the micro-plate reader (BioTek, Winooski, VT, USA). The following formula was used to calculate the bacterial growth inhibition (INH%):

$$\mathrm{INH}\%\;=\;\frac{100-({\mathrm{OD}}_{\;630\;\mathrm{sample}}\times 100)}{{\mathrm{OD}}_{\;630\;\mathrm{control}}}$$

### 3.6. Anti-Thrombolytic Activity

The anti-thrombolyticactivity of newly synthesized products was studied using the previously reported method [26].The venous blood samples were taken from healthy human volunteers and shifted to sterile eppendorf tubes/microcentrifuge tubes. Five hundred microliters of blood containing microcentrifuge tubes were incubated for the period of 45 min at 37 °C temperature to form clots. After clot formation, the microcentrifuge tubes, without serum which was discarded earlier, were filled with 100 μL solution of test samples (**2a**–**i**). After this addition, all eppendorf tubes were again incubated for 90 min at 37 °C and observed. For standard clot lysis agent, the thrombolytic drug streptokinase was used while in case of negative thrombolytic control, and water was used in each assay. The conclusions for clot lysis activity were determined as percentages.

## 4. Conclusions

The present study reports the Suzuki-Miyaura cross-coupling reaction for the synthesis of a series of 5-aryl-2-bromo-3-hexylthiophene (**2a**–**i**). TLC was used to examine the progress of reaction and purity of all new products, and their structure elucidation was confirmed by spectroscopic techniques. The detailed investigation shows that aryl rings possessing different electron-withdrawing and electron-donating groups have appreciable effects on the haemolytic, biofilm inhibition and anti-thrombolytic activities of the newly synthesized compounds. Excellent haemolytic, biofilm inhibition and anti-thrombolytic actions were exhibited by compounds with electron-withdrawing groups on the aryl ring. The positions and natures of functional groups on aryl rings were compared with each other to investigate the efficiency of various 5-aryl-2-bromo-3-hexylthiophene (**2a**–**i**) derivatives. The compound 2-bromo-5-(3-chloro-4-fluorophenyl)-3-hexylthiophene (**2g**) was found to be most efficient and active against bacteria and revealed superior haemolytic as well as biofilm inhibition activities, while other compounds showed mild to moderate activities. In addition, 2-bromo-3-hexyl-5-(4-iodophenyl)thiophene (**2e**) also exhibited highest anti-thrombolytic activity, indicating the usefulness of these newly synthesized compounds for medicinal applications.
